# The impact of stigma on post-traumatic growth in patients with enterostomy: the chain mediating effect of coping styles and self-care

**DOI:** 10.3389/fpsyt.2026.1891377

**Published:** 2026-08-10

**Authors:** Lulu Ma, Ying Piao, Yu Zhou, Xiaohuan Wang

**Affiliations:** 1 Department of General Surgery, Northern Theater Command General Hospital of the People’s Liberation Army, Shenyang, Liaoning, China; 2 Wound and Stoma Clinic, The First Affiliated Hospital of China Medical University, Shenyang, Liaoning, China; 3 Department of Stoma Clinic of the General Surgery, Northern Theater Command General Hospital of the People’s Liberation Army, Shenyang, Liaoning, China

**Keywords:** chain mediating effect, coping styles, enterostomy, post-traumatic growth, self-care, stigma

## Abstract

**Objective:**

This study aimed to investigate whether coping styles and self-care competence sequentially mediate the association between perceived stigma and post-traumatic growth among patients undergoing enterostomy.

**Methods:**

This was a retrospective cross-sectional study. A total of 240 patients with enterostomies who were admitted to our hospital between January 2024 and June 2025 were selected. The clinical data of these patients were extracted from the hospital’s electronic medical record system, including the General Information Questionnaire, Social Impact Scale (SIS), Medical Coping Modes Questionnaire (MCMQ), Exercise of Self-Care Agency Scale (ESCA), and Post-Traumatic Growth Inventory (PTGI). Data analysis was performed using SPSS 26.0 software and the PROCESS macro(Model 6); regression-based path analysis was used to test the hypothesized chain mediating pathway.

**Results:**

The total stigma score of the 240 patients with enterostomy was (59.54 ± 12.66) points; the scores of confrontation, avoidance, and resignation were (17.91 ± 4.24), (15.19 ± 3.58), and (11.25 ± 3.21) points, respectively; the total self-care score was (63.52 ± 5.50) points; and the total post-traumatic growth score was (53.36 ± 6.10) points. Stigma in patients with enterostomy was negatively correlated with confrontation, self-care, and post-traumatic growth (*r* = -0.502, -0.430, -0.472, *P* < 0.05), and positively correlated with avoidance and resignation (*r* = 0.563, 0.482, *P* < 0.05). Stigma had a significant negative total effect on post-traumatic growth (unstandardized *β* = -0.930, *t* = -8.268, *P* < 0.001). After controlling for mediating variables, stigma still had a significant direct negative effect on post-traumatic growth. The serial chain mediating effect of “stigma→confrontation→self-care→post-traumatic growth” was significant. The total indirect effect was β=-0.529, which accounted for 63.75% of the total effect. The overall model had an R² of 0.265 (*F* = 38.642, *P* < 0.001), indicating that the model explained 26.5% of the variance in post-traumatic growth.

**Conclusion:**

Patients with colostomies commonly experience perceived stigma. Stigma influences post-traumatic growth through both direct and sequential indirect pathways. Specifically, higher levels of perceived stigma are associated with reduced use of confrontational coping strategies, which subsequently diminish self-care ability and ultimately hinder post-traumatic growth.

## 1 Introduction

Enterostomy is a life-saving surgical procedure widely used in the management of various intestinal disorders, including colorectal cancer, intestinal obstruction, and perforation. This procedure involves creating an artificial anus on the abdominal wall by exteriorizing the proximal segment of the intestine, allowing fecal excretion and preserving vital physiological functions (1). Epidemiological data indicate that approximately 100,000 new patients undergo enterostomy in China each year, with the total number of living patients with enterostomy exceeding 1 million (2). Although enterostomy is critical for patient survival, it permanently alters normal defecation patterns, resulting in significant changes in body image and disruption of daily activities. Compounded by limited public understanding and acceptance of enterostomy, these changes often lead to the development of stigma—a distressing psychological state characterized by feelings of shame, inferiority, and self-blame, as well as fear of social rejection and discrimination (3).

Post-traumatic growth (PTG) refers to the positive psychological changes that individuals may experience following highly challenging or traumatic life circumstances, including a greater appreciation of life, recognition of new possibilities, enhanced personal strength, improved interpersonal relationships, and spiritual development (4). Unlike resilience, which is a return to baseline functioning, PTG represents forward movement beyond pre-trauma adaptation. In the context of enterostomy, PTG may manifest as patients developing a deeper appreciation for health, discovering personal strengths through the process of ostomy self-management, building more meaningful connections with others who share similar experiences, and finding a renewed purpose in life. Research has shown that stigma is an important negative factor affecting post-traumatic growth. The stronger the stigma of patients with enterostomy, the lower the level of post-traumatic growth (5). However, the mechanism of action between the two has not been fully elucidated.

Coping styles are cognitive and behavioral strategies adopted by individuals when they face stressful events. The Medical Coping Modes Questionnaire (MCMQ) categorizes coping into three types: confrontation (actively facing and dealing with the stressor), avoidance (attempting to ignore or escape from the stressor), and resignation (giving up and accepting the situation helplessly). When patients with enterostomy face traumas, such as changes in body image and abnormal defecation function, different coping styles affect their psychological state and behavioral performance (6).

Self-care competence is the core of postoperative rehabilitation for patients with enterostomy, referring to the ability of patients to independently complete nursing operations, such as ostomy bag replacement, cleaning, and complication prevention, under the guidance of medical staff (7). Based on Orem’s Self-Care Deficit Theory, self-care competence encompasses the knowledge, skills, and motivation required to perform self-care behaviors. Good self-care ability not only reduces ostomy-related complications but also enhances patients’ self-efficacy and promotes psychological rehabilitation. Theoretically, stigma may affect self-care ability by influencing coping styles, ultimately affecting post-traumatic growth. Zhang and Li (2025) (8) examined similar variables (stigma, coping styles, self-care, and PTG) among colorectal cancer patients with permanent enterostomy and found significant associations. However, the present study extends this work by specifically testing the serial chain mediating pathway (stigma→confrontation→self-care→PTG) using PROCESS Model 6, which allows for a more nuanced understanding of the sequential mechanism compared to the parallel mediation approach used in previous studies. Additionally, our study included a broader spectrum of patients with enterostomy (including both colostomy and ileostomy, and mixed etiologies), which may enhance the generalizability of the findings. Currently, few studies have examined the relationship between stigma and in patients with enterostomy, and most studies have only explored a single mediating mechanism, lacking an in-depth analysis of the chain effect of multiple variables.

## 2 Materials and methods

### 2.1 Research subjects

A retrospective cross-sectional study design was adopted. 240 patients with enterostomy who were admitted to our hospital from January 2024 to June 2025 were selected. The clinical data of these patients were extracted from the hospital’s electronic medical record system. This study was approved by the Medical Ethics Committee of our hospital. The enrolled patients had heterogeneous clinical characteristics, including different primary intestinal diseases, two types of ostomy and a wide postoperative follow-up interval of 1–12 months. To eliminate the confounding influence of sample heterogeneity on the mediating paths, all regression models in this study adjusted the above clinical covariates simultaneously.

The inclusion criteria were as follows: ① Age ≥18 years old, conscious, able to communicate normally and understand the content of the questionnaire; ② Underwent enterostomy surgery for ≥1 month, with good ostomy healing with no serious complications (such as ostomy necrosis, severe infection, parastomal hernia, etc.); and ③ Signed informed consent. The exclusion criteria were as follows: ① Complicated with severe heart, liver, or kidney dysfunction or distant metastasis of malignant tumors; ② Previous history of mental illness or taking antipsychotic drugs; and ③ Simultaneously participation in other psychological intervention studies.

Sample size estimation: According to the empirical rule of sample size estimation proposed by Kline (9), the sample size for path analysis should be at least 10–20 times the number of estimated parameters. This study involved independent variables (stigma), mediating variables (coping styles, self-care ability), dependent variables (post-traumatic growth) and control variables, requiring the estimation of approximately 12–15 parameters, with a minimum sample size requirement of 150–300 cases. Meanwhile, referring to the sample size recommendations for chain mediating effects by Fang et al. (10), Monte Carlo simulations found that when the expected mediating effect is small (β≈0.14), the sample size required to achieve 80% statistical power is approximately 200 cases. Considering the possible missing data in retrospective studies, 240 cases were finally included in this study, meeting the statistical requirements.

### 2.2 Research tools

#### 2.2.1 General information questionnaire

The questionnaire was self-designed to collect demographic and clinical characteristics, including patient age, gender, education level, marital status, occupation, ostomy type (colostomy/ileostomy), monthly family income, and time since ostomy surgery (1–3 months, 3–6 months, and 6–12 months).

#### 2.2.2 Social impact scale

The Social Impact Scale (SIS), developed by Wright-Fife et al., was used to assess perceived stigma among patients with chronic diseases (11) The Chinese version was revised by Pan et al. (12), consisting of 24 items and four dimensions: social exclusion, economic insecurity, internalized shame, and social isolation. Each item uses a 4-point Likert scale (1=strongly disagree to 4=strongly agree), with a total score ranging from 24 to 96. The Cronbach’s α coefficient of the scale in this study was 0.89. McDonald’s ω was 0.91.

#### 2.2.3 Medical coping modes questionnaire

MCMQ was compiled by Feifel et al. (13) and localized by Shen et al. (14). It consists of 20 items divided into three dimensions: confrontation, avoidance, and resignation. Each item uses a 4-point Likert scale. Higher scores in each dimension indicate that patients are more inclined to adopt that coping style. The Cronbach’s α coefficients of the three dimensions of the scale were 0.821, 0.785, and 0.763. respectively. McDonald’s ω values were 0.83, 0.79, and 0.77, respectively.

#### 2.2.4 Exercise of self-care agency scale

ESCA was compiled with reference to the ESCA scale (15)(28-item version). It consists of 28 items, including care skills, self-concept, health knowledge level, and self-care responsibility. Each item uses a 5-point Likert scale(0=very poor to 4=very good), with a total score ranging from 0 to 112. The Cronbach’s α coefficient of the scale in this study was 0.91.McDonald’s ω was 0.92.

#### 2.2.5 Post-traumatic growth inventory

PTGI was compiled by Tedeschi et al. (16) and localized by Wang et al. (17).)t consists of 21 items divided into five dimensions: appreciation of life, new possibilities, personal strength, relating to others, and spiritual change. Each item uses a 6-point Likert scale (0=not at all to 5=to a very great degree), with a total score ranging from 0 to 105. Higher scores indicate a higher level of post-traumatic growth. The Cronbach’s α coefficient of the scale in this study was 0.94, and McDonald’s ω was 0.95.

### 2.3 Data collection method

Two research nurses who received unified training conducted questionnaire surveys when patients returned to the hospital for reexamination or during home follow-up 1–3 months after surgery. Before the survey, the purpose, significance, and filling method of the study were explained to the patients, with emphasis on anonymity and confidentiality. After the patients signed informed consent forms, the questionnaires were distributed on-site and collected immediately. For patients with a low education level or reading difficulties, the investigators read each item aloud and filled in the answers on behalf of the patients after the patients responded. A total of 245 questionnaires were distributed, and 240 valid questionnaires were recovered, with an effective recovery rate of 97.96%. All questionnaires were double-checked and entered, and invalid questionnaires with regular answers or missing items >10% were excluded.

Timing of data collection: Patients were surveyed during follow-up visits at 1–12 months post-surgery. This time range was chosen because the first year after ostomy creation is a critical period for psychological adjustment to ostomy. Time since surgery was included as a covariate in the analyses because it may be associated with both self-care competence and PTG. The distribution of participants by postoperative period was: 1–3 months (36.25%), 3–6 months (38.75%), and 6–12 months (25.00%).

### 2.4 Statistical methods

SPSS 26.0 statistical software and the PROCESS plugin (version 3.5) were used for data analysis. Measurement data are described as mean ± standard deviation, and count data are described as number (percentage). The Shapiro-Wilk test was used for the normality test of measurement data, and the variance inflation factor (VIF) was used to test multicollinearity. VIF<5 indicates no serious collinearity. Pearson’s correlation analysis was used to explore the correlation between variables for measurement data conforming to a normal distribution. Model 6 in the PROCESS plugin (a regression-based path analysis tool that works with observed composite scores) was used to test the chain mediating effect of coping styles and self-care on the impact of stigma on post-traumatic growth. When constructing the serial multiple mediation model via PROCESS Model 6, we fully adjusted for key demographic and clinical covariates that may lead to sample heterogeneity, including age, gender, education level, ostomy type (colostomy/ileostomy, categorical variable), postoperative time (1–3 months/3–6 months/6–12 months), and primary disease etiology. All path coefficients, direct and indirect effect values reported in **Table 1** were calculated after controlling the above confounding factors. The Bootstrap method (5,000 repeated samplings) was used to calculate the 95% confidence interval (CI). If the CI does not contain 0, the mediating effect is established. To address potential common method variance, Harman’s single-factor test was conducted. *P* < 0.05 was considered statistically significant. This study followed the STROBE guidelines for cross-sectional studies. To control for common method bias caused by self-reported questionnaires collected at a single time point, Harman’s single-factor test was conducted for all scale items. All items were subjected to unrotated exploratory factor analysis; if the maximum variance explained by the first extracted common factor was less than 40%, the common method bias was considered negligible.

**Table 1:** Mediating effect test of stigma on post-traumatic growth.

Effect	Path relationship	Unstandardized effect value	*t*	*P*	*SE*	Bootstrap 95% *CI*		Standardized effect value	Effect proportion (%)
						Boot *LLCI*	Boot *ULCI*		
Direct effect	Stigma→Post-traumatic growth	-0.337	-3.183	0.002	0.121	-0.546	-0.128	-0.171	36.25
Mediating effect	Stigma→Confrontation→Post-traumatic growth	-0.136	–	–	0.060	-0.270	-0.038	-0.069	14.6
	Stigma→Avoidance→Post-traumatic growth	-0.156	–	–	0.060	-0.290	-0.053	-0.079	16.8
	Stigma→Resignation→Post-traumatic growth	-0.026			0.037	-0.086	-0.016	-0.013	2.8
	Stigma→Self-care→Post-traumatic growth	-0.156			0.061	-0.295	-0.055	-0.079	16.8
	Stigma→Confrontation→Self-care→Post-traumatic growth	-0.055			0.027	-0.116	-0.013	-0.028	5.9
	Stigma→Avoidance→Self-care→Post-traumatic growth	-0.045			0.024	-0.102	-0.010	-0.023	4.8
	Stigma→Resignation→Self-care→Post-traumatic growth	-0.012			0.011	-0.038	-0.001	-0.006	1.3
Total indirect effect	**-**	-0.529	–	–	–	–	–	–	63.75
Total effect	Stigma→Post-traumatic growth	-0.930	-8.268	<0.001	0.113	-1.152	-0.708	-0.472	100.00

The significance of the mediating effect is based on Bootstrap 95% CI; CI not containing 0 indicates a significant effect; the direct effect is tested by t-test. All direct and indirect effect values in this table were calculated after adjusting for covariates including age, gender, education level, ostomy type, postoperative time and primary disease etiology.

## 3 Results

### 3.1 General information of patients

A total of 240 patients with enterostomies were included in this study. Among them, 135 (56.25%) were male and 105 (43.75%) were female, with a mean age of 58.64 ± 11.32 years (range, 25–86). Regarding educational attainment, 62 (25.83%) had primary school education or below, 80 (33.33%) had completed junior high school, 58 (24.17%) had completed high school or technical secondary school, and 40 (16.67%) had attained a junior college education or higher. Colostomy was performed in 142 patients (59.17%) and ileostomy in 98 (40.83%). The time since ostomy surgery was 1–3 months in 87 patients (36.25%), 3–6 months in 93 (38.75%), and 6–12 months in 60 (25.00%).

### 3.2 Scores of each variable

The total stigma score of 240 patients with enterostomy was (59.54 ± 12.66) points. The scores of confrontation, avoidance, and resignation were (17.91 ± 4.24), (15.19 ± 3.58), and (11.25 ± 3.21) points, respectively. The total self-care score was (63.52 ± 5.50) points and the total post-traumatic growth score was (53.36 ± 6.10) points, as shown in **Table 2**.

**Table 2:** Scores of each variable ( $\bar{x}$ ± s, points).

Item	Score
Total stigma score	59.54 ± 12.66
Social exclusion	21.56 ± 5.25
Economic insecurity	6.75 ± 2.14
Internalized shame	12.36 ± 3.10
Social isolation	18.80 ± 3.09
Coping style scores	
Confrontation	17.91 ± 4.24
Avoidance	15.19 ± 3.58
Resignation	11.25 ± 3.21
Total self-care score	63.52 ± 5.50
Care skills	23.01 ± 3.96
Self-concept	18.45 ± 2.52
Health knowledge level	13.81 ± 2.99
Self-care responsibility	8.12 ± 1.33
Total post-traumatic growth score	53.36 ± 6.10
Appreciation of life	18.56 ± 3.25
New possibilities	9.80 ± 2.09
Personal strength	8.54 ± 1.16
Relating to others	9.36 ± 2.10
Spiritual change	8.56 ± 1.20

### 3.3 Correlation analysis between variables

Stigma in patients with enterostomy was negatively correlated with confrontation, self-care, and post-traumatic growth (*r* = -0.502, -0.430, -0.472, *P* < 0.05), and positively correlated with avoidance and resignation (*r* = 0.563, 0.482, *P* < 0.05), as shown in **Table 3**. The VIF values of all variables were <5, indicating no serious multicollinearity that could be included in the regression model.

**Table 3:** Correlation analysis of each variable.

Variable	Stigma	Confrontation	Avoidance	Resignation	Self-care	Post-traumatic growth
Stigma	1	–	–	–		
Confrontation	-0.502^**^	1	–	–		
Avoidance	0.563^**^	-0.368^**^	1	–		
Resignation	0.482^**^	-0.495^**^	0.551^**^	1		
Self-care	-0.430^**^	0.266^**^	-0.230^**^	-0.150^*^	1	
Post-traumatic growth	-0.472^**^	0.303^**^	-0.338^**^	-0.357^**^	0.423^**^	1

^*^P < 0.05; ^**^P < 0.001.

### 3.4 Test of common method bias

Harman’s single-factor test was performed on all items of SIS, MCMQ, ESCA and PTGI. Unrotated exploratory factor analysis extracted a total of 13 common factors with eigenvalues greater than 1. The variance interpretation rate of the first common factor was 28.60%, which was far lower than the critical threshold of 40%. It indicated that there was no serious common method bias in the self-reported data of this study, and the reliability of subsequent correlation and mediation analysis results would not be interfered.

### 3.5 Common method bias test

Harman’s single-factor test was conducted to assess common method variance. The first unrotated factor accounted for 28.6% of the total variance, which is below the recommended threshold of 40%, indicating that the common method bias was not a significant concern in this study.

### 3.6 Chain mediating effect test

After controlling for age, gender, education, ostomy type, postoperative duration and primary disease etiology, the serial multiple mediation model based on PROCESS Model 6 was constructed with stigma as independent variable, PTG as dependent variable, and three coping dimensions + self-care as parallel mediators.

Considering stigma as the independent variable, post-traumatic growth as the dependent variable, coping styles, and self-care as the mediating variables, the mediating effect test was conducted using PROCESS Model 6. Three coping dimensions (confrontation, avoidance, and resignation) and self-care were entered as parallel mediators in the following order: stigma → coping dimensions → self-care → PTG. The results showed that stigma had a significant negative total effect on post-traumatic growth. After controlling for mediating variables, stigma still had a significant direct negative effect on post-traumatic growth (*β* = -0.171, 95% *CI*: -0.546 to -0.128), indicating that stigma was directly associated with lower PTG. The direct effect accounted for 36.25% of the total effect, suggesting that stigma primarily operates through indirect pathways.

**Figure 1:**
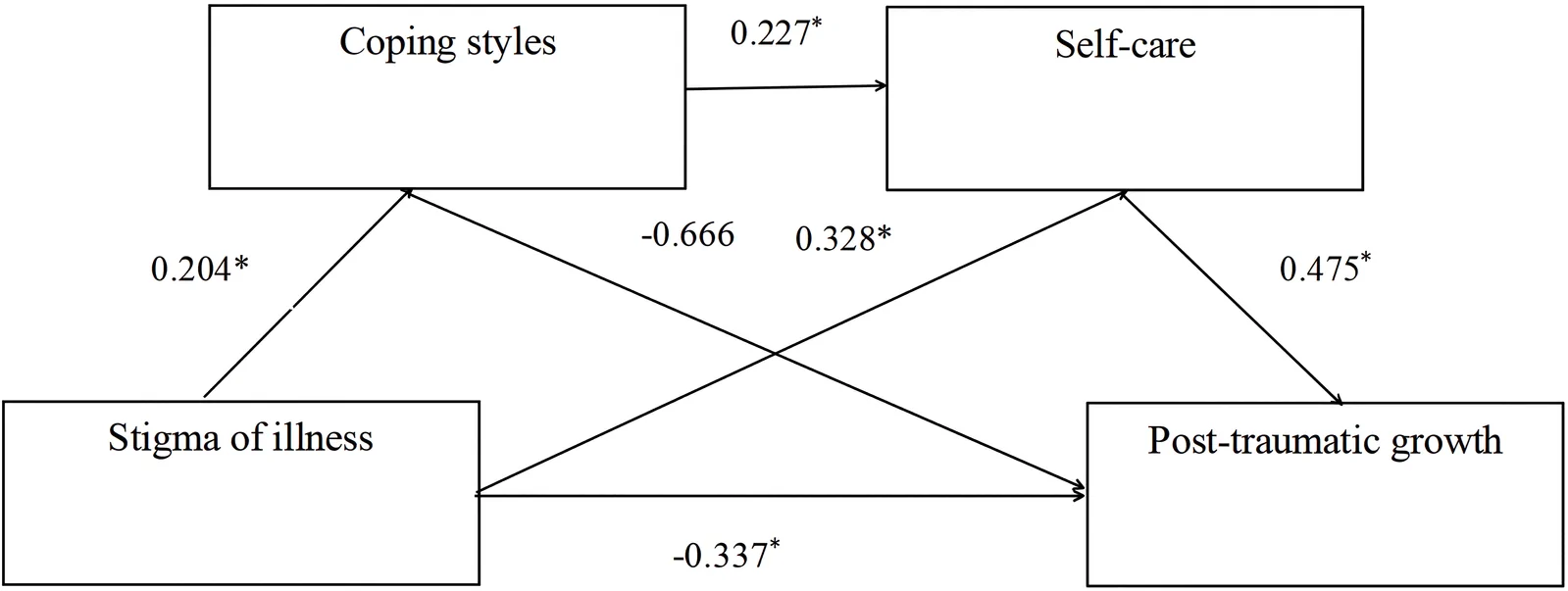
Chain model of the mediating effect of stigma on post-traumatic growth. *P* < 0.05. *P<0.05.

The serial chain mediating effect of stigma → confrontation → self-care → post-traumatic growth was statistically significant (unstandardized β = −0.055, bootstrap 95% CI: −0.116 to −0.013), accounting for 5.9% of the total effects. Although the total indirect effect accounted for 63.75% of the total effect, this reflected the combined contributions of all five indirect pathways. The serial chain pathway itself contributed the smallest proportion among the significant indirect effects and, therefore, should not be interpreted as the dominant mediating pathway. The overall model explained 26.5% of the variance in post-traumatic growth (R² = 0.265, F = 38.642, P < 0.001). All path coefficients are shown in **Figure 1**, and the mediation model was estimated after adjusting for age, sex, education level, ostomy type, and time since the surgery.

## 4 Discussion

### 4.1 The relationship between stigma and post-traumatic growth in patients with enterostomy

The results of this study showed that the total stigma score of 240 patients with enterostomy was (59.54 ± 12.66) points. With the SIS total score range of 24-96 (theoretical midpoint = 60), this indicates a moderate level of stigma. This finding is comparable to the results of similar studies in China (18, 19). This indicates that patients generally experience obvious stigma after enterostomy surgery. Several factors may explain these findings. First, enterostomy alters the normal mode of defecation, resulting in impaired body image. Second, limited public awareness and understanding of enterostomy may cause patients to fear discrimination, stigmatization, and social exclusion. Third, the complexity of ostomy care, together with concerns about odor and leakage, may further exacerbate feelings of inferiority, embarrassment, and shame (20). Meanwhile, this study found that the total post-traumatic growth score of patients was (53.36 ± 6.10) points, which was at a moderate level, suggesting that some patients can experience positive psychological changes after experiencing the traumatic event of enterostomy, such as re-understanding the meaning of life and enhancing their personal strength.

Correlation analysis showed that stigma was significantly negatively correlated with PTG and had a significant direct negative effect on PTG, which is consistent with previous research conclusions (21, 22). As a strong negative psychological experience, stigma is associated with lower PTG through multiple pathways. First, stigma may lead to self-denial and self-deprecation in patients who think they are “incomplete” and “defective,” making it difficult to find positive meaning from traumatic events. Second, stigma may promote patients to adopt social avoidance behaviors, reducing communication and interaction with others, cutting off channels for obtaining social support, which is an important factor promoting post-traumatic growth. Finally, long-term stigma may consume patients’ psychological resources, leading them to experience negative emotions such as anxiety and depression, and making it impossible to carry out positive psychological adjustment and growth (23).

### 4.2 Mediating effect of coping styles between stigma and post-traumatic growth

The results of this study showed that coping styles played a partial mediating role between stigma and PTG, among which the path of “stigma→confrontation→post-traumatic growth” was significant, as were the separate mediating effects of avoidance and resignation (both Bootstrap 95% CIs excluded zero). This indicates that stigma is associated with lower PTG through all three coping styles. Confrontational coping is a positive coping strategy that refers to the behavioral tendency of individuals to actively face problems, seek solutions, and seek social support (24). This study found that stigma was significantly negatively correlated with confrontational coping; that is, patients with stronger stigma were more inclined to avoid problems rather than actively face them. The mechanism is that stigma causes strong shame and fear in patients. To avoid experiencing this pain again, patients subconsciously avoid topics, scenes, and social activities related to ostomy, thus reducing the opportunity to adopt confrontational coping (25). Confrontational coping was significantly and positively correlated with PTG. This is because actively facing problems can help patients better adapt to the life changes brought by ostomy, enhance self-efficacy, and discover their own potential and strength in the process of solving problems, thereby promoting PTG.

Although avoidance and resignation coping were significantly positively correlated with stigma and negatively correlated with PTG, their separate mediating effects were not significant. This may be because avoidance and resignation coping mainly act on PTG indirectly by influencing other variables (such as self-care ability) rather than directly influencing it. In addition, the relatively low scores of avoidance and resignation coping in this study may also be one of the reasons for the insignificant mediating effect.

The separate mediating effects of avoidance and resignation were also significant in this study (Bootstrap CIs: -0.290 to -0.053 for avoidance; -0.086 to -0.016 for resignation). This finding suggests that avoidance and resignation coping styles, which are more passive and maladaptive, may be activated by stigma and are subsequently associated with lower PTG.

### 4.3 Mediating effect of self-care and chain mediating effect

The most important finding of this study is that the serial chain mediating effect of “stigma→confrontation→self-care→post-traumatic growth” was significant. This indicates that stigma is associated with lower PTG not only directly or through individual mediators but also through a sequential pathway. Higher stigma is associated with reduced confrontational coping, which, in turn, is associated with lower self-care competence, which is ultimately associated with reduced PTG. Self-care ability is the core of postoperative rehabilitation for patients with enterostomy, which is not only related to the prevention of ostomy-related complications but also has an important impact on patients’ psychological state (26). The results of this study showed that stigma was significantly negatively correlated with self-care ability, confrontational coping was significantly positively correlated with self-care ability, and self-care ability was significantly positively correlated with PTG. The chain mechanism of action is as follows: patients with stronger stigma are more inclined to adopt avoidant coping styles and are unwilling to actively learn ostomy care knowledge and skills, leading to decreased self-care ability. Insufficient self-care ability makes patients more prone to ostomy complications, aggravates physical discomfort and psychological burden, reduces patients’ self-efficacy and quality of life, and may influence the generation of PTG. In contrast, patients with lower stigma are more willing to adopt confrontational coping styles, actively learn ostomy care skills, have stronger self-care ability, can better manage ostomy, reduce the occurrence of complications, enhance their sense of control over life, and promote PTG. A Turkish study pointed out that there was a moderate positive correlation between social support and ostomy self-efficacy, providing empirical support for the path of “social support→self-care” (27).

This study also found that the separate mediating effect of “stigma→self-care→post-traumatic growth” was significant, indicating that stigma can also directly affect patients’ self-care ability and act on PTG. This may be because stigma may influence patients’ self-abandonment psychology, who think they cannot complete ostomy care, reducing their motivation and willingness to carry out self-care.

### 4.4 Clinical significance and research limitations

First, clinical medical staff should attach great importance to the stigma problem of enterostomy patients, take stigma assessment as a routine nursing content after surgery, and timely identify patients with high levels of stigma. Methods such as providing ostomy knowledge education, organizing ostomy patient associations, and inviting successfully rehabilitated patients to share their experiences can help patients correctly understand ostomy and reduce stigma. Second, attention should be paid to cultivating patients’ positive coping styles, especially their confrontational coping ability. Methods such as cognitive behavioral intervention and psychological counseling can help patients identify and change negative cognitive and behavioral patterns, guide them to face the life changes brought by ostomy squarely, and actively seek social support and professional help. Finally, self-care guidance for patients should be strengthened to improve their self-care abilities. A variety of methods, such as one-on-one demonstration, operation practice, and video teaching, can be used to help patients master skills such as ostomy bag replacement, ostomy cleaning, and complication prevention.

This study has several limitations. First, the cross-sectional design precludes conclusions regarding the causality or temporal ordering of the proposed mediation pathways. Although the hypothesized sequence (stigma → coping → self-care → post-traumatic growth [PTG]) is theoretically supported, longitudinal studies are required to verify these causal relationships. Second, this was a single-center study with a moderate sample size, which may limit the generalizability of our findings. Third, all variables were assessed using self-report questionnaires and were therefore susceptible to recall and social desirability biases. Nevertheless, Harman’s single-factor test suggested that common method variance was unlikely to have substantially influenced the results. Fourth, other potentially important mediators or moderators, such as social support, self-efficacy, resilience, and coping flexibility, were not examined in this study. Future studies should incorporate these factors to provide a more comprehensive understanding of the mechanisms linking stigma and PTG in this population. Fifth, objective clinical outcomes, such as stoma-related complications and hospital readmissions, were not included to complement self-reported data. Integrating subjective and objective outcome measures in future research would provide a more robust evaluation. Finally, although the 28-item version of the Exercise of Self-Care Agency (ESCA) Scale has demonstrated acceptable reliability and validity, its use may limit comparability with studies employing the original 43-item version of the scale. Future studies should consider using the full version or provide a clear rationale for selecting the abbreviated version. In addition, although we adjusted multiple heterogeneous clinical covariates such as ostomy classification and postoperative time in the mediation model, there were still unmeasured potential confounding variables that could not be excluded.

In conclusion, patients with colostomies generally experience varying degrees of perceived stigma. Stigma influences posttraumatic growth (PTG) through both direct and indirect pathways. Specifically, higher levels of perceived stigma are associated with reduced use of confrontational coping strategies, which subsequently impairs self-care ability and hinders post-traumatic growth.

## Data Availability

The original contributions presented in the study are included in the article/supplementary material. Further inquiries can be directed to the corresponding author.

## References

[1] Rodriguez Silva JA Maykel JA . Loop ileostomy reversal. Colorectal Dis. (2023) 25:160. doi: 10.1111/codi.16287 35901219

[2] Portenkirchner C Turina M Rickenbacher A . Single incision laparoscopic surgery (SILS) versus conventional laparoscopic technique for ileostomy: a retrospective cohort study. Langenbecks Arch Surg. (2022) 407:1757–63. doi: 10.1007/s00423-022-02473-0 35639135

[3] Roicke A Esser P Hornemann B Ernst J . Schmerzbedingte Stigmatisierung bei Patienten mit Brust-, Darm-, Prostata- oder Lungenkrebs : Ergebnisse einer bizentrischen registerbasierten Querschnittstudie [Pain-related stigma in patients with breast, colon, prostate or lung cancer : Results of a bicentric register-based cross-sectional study. Schmerz. (2024) 38:390–9. doi: 10.1007/s00482-023-00752-3 37710022 PMC11576764

[4] Dell'Osso L Carpita B Nardi B Bonelli C Calvaruso M Cremone IM . Biological correlates of post-traumatic growth (PTG): a literature review. Brain Sci. (2023) 13:305. doi: 10.3390/brainsci13020305 36831848 PMC9953771

[5] Huang B Liu G Huang J He S Li W Xiao S . The impact of discharge readiness on post-traumatic growth in patients after thyroid cancer surgery: the mediating role of sickness-related stigma. Front Oncol. (2024) 14:1361036. doi: 10.3389/fonc.2024.1361036 39286012 PMC11403406

[6] Knowles SR Tribbick D Connell WR Castle D Salzberg M Kamm MA . Exploration of health status, illness perceptions, coping strategies, psychological morbidity, and quality of life in individuals with fecal ostomies. J Wound Ostomy Continence Nurs. (2017) 44:69–73. doi: 10.1097/WON.0000000000000295 28060005

[7] Sayar S Vural F . Effects of a support group intervention on adaptation to ostomy, quality of life, and complication severity in patients with an ostomy. J Wound Ostomy Continence Nurs. (2025) 52:485–91. doi: 10.1097/WON.0000000000001226 41235849

[8] Zhang M Li Y . Relationships between stigma, coping styles, self-care and post-traumatic growth among colorectal cancer patients with permanent enterostomy: a cross-sectional study. Heliyon. (2025) 11:e38902. doi: 10.1016/j.heliyon.2024.e38902 38826717

[9] Kline RB . Principles and Practice of Structural Equation Modeling (4th Ed.). New York: Guilford Press (2016).

[10] Fang J Wen ZL Zhang MQ . Multilevel mediating effect analysis based on structural equation modeling. Adv Psyc Sci. (2014) 22:154–63. doi: 10.3724/SP.J.1042.2014.00530

[11] Wright FER . The dimensionality of stigma: a comparison of its impact on the self of persons with HIV/AIDS and cancer. J Heal Soc Behav. (2000) 41:50–67. doi: 10.2307/2676360 10750322

[12] Pan AW Chung L Fife BL Hsiung PC . Evaluation of the psychometrics of the Social Impact Scale: a measure of stigmatization. Int J Rehabil Res. (2007) 30:235–8. doi: 10.1097/mrr.0b013e32829fb3db 17762770

[13] Feifel H Strack S Tong N . Coping strategies and associated features of medically ill patients. Psychosom Med. (1987) 49:616–25. doi: 10.1097/00006842-198711000-00007 3423168

[14] Shen XH Jiang QJ . Chinese version of the medical coping style questionnaire - test report of 701 cases. Chin Behav Med Sci. (2000) 9:22–4. doi: 10.3760/cma.j.issn.1674-6554.2000.01.008 30704229

[15] Wang HH Laffrey SC . Preliminary development and testing of instruments to measure self-care agency and social support of women in Taiwan. Kaohsiung J Med Sci. (2000) 16:459–67. doi: 10.1037/t05545-000 11271731

[16] Tedeschi RG Calhoun LG . The posttraumatic growth inventory: measuring the positive legacy of trauma. J Trauma Stress. (1996) 9:455–71. doi: 10.1002/jts.2490090305 8827649

[17] Wang J . Development of the post-traumatic growth rating scale and norms for individuals experiencing accidental trauma. Second Milit Med Univer (2011). [D].

[18] Ding X Chen L Xue Y Li HY Ding YY . Factors influencing stigma in elderly patients with colorectal cancer after surgery who have undergone intestinal stoma construction and the construction of a predictive model. J Psyc. (2024) 37:421–5. doi: 10.3969/j.issn.2095-9346.2024.04.016

[19] Li J Wang XS Li QJ Li YJ Wang QW Guo J . The effect of narrative nursing on reducing the stigma, adapting to the stoma, and improving the quality of life of patients with permanent intestinal stoma. Chin J Mod Nurs. (2024) 30:4889–95. doi: 10.3760/cma.j.cn115682-20240105-00106 30704229

[20] Gros HL Taha-Mehlitz S Erdem S Worni M Henschel M Huber AK . Listening to patients: a qualitative study on diagnostic delay, coping strategies and stigma in early-onset colorectal cancer. Colorectal Dis. (2025) 27:e70285. doi: 10.1111/codi.70285 41146579 PMC12559872

[21] Wei D Wang X Wang M Wang J Chen F Jin L . Correlated factors of posttraumatic growth in patients with colorectal cancer: a systematic review and meta-analysis. Int J Nurs Sci. (2024) 12:96–105. doi: 10.1016/j.ijnss.2024.12.004 39990990 PMC11846582

[22] Karahan A Çıtak EA Çevik B Abbasolu A Uurlu Z . Determining the perception of stigma of individuals with ostomies. BMC Psychol. (2025) 13:767. doi: 10.1186/s40359-025-03112-1 40640969 PMC12247216

[23] García-Torres F Gómez-Solís Á Castillo-Mayén R Espejo-Siles R Jurado-González F Jablonski M . Psychological flexibility and its correlations with and predictive utility for emotional wellbeing, fatigue, insomnia, and post-traumatic growth in cancer patients undergoing treatment. Front Psychol. (2026) 17:1768998. doi: 10.3389/fpsyg.2026.1768998 41909564 PMC13017947

[24] Levenets O Stepurko T Polese A Pavlova M Groot W . Coping strategies of cancer patients in Ukraine. Int J Health Plann Manage. (2019) 34:1423–38. doi: 10.1002/hpm.2802 31095792

[25] Goldberg N Appelbaum M Rudnicki Y Soback H Rahmani A Avital S . Patients refusing a permanent colostomy for rectal cancer: clinical outcome and psychological aspects. Support Care Cancer. (2025) 33:774. doi: 10.1007/s00520-025-09837-4 40782212 PMC12335384

[26] Lisboa CR Spira JAO Borges EL . Self-care concept for people with elimination ostomy: a scoping review. Rev Esc Enferm Usp. (2024) 58:e20240041. doi: 10.1590/1980-220X-REEUSP-2024-0041en 39761323 PMC11702974

[27] Aker FZ Karazeybek E . Relationship between perceived social support and stoma self-efficacy in permanent colostomy patients: a correlational study. J Eval Clin Pract. (2025) 31:e14117. doi: 10.1111/jep.14117 39099203 PMC11656665

